# How to Arrange for Scattered Areas

**Published:** 1919-10-04

**Authors:** 


### MUNICIPAL MEDICAL SERVICES.

### How to Arrange for Scattered Areas.

The State has imposed upon local authorities far-reaching powers for establishing new and extending old medical services. Administration is easy in big centres but difficult in scattered areas, and a report of Dr. J. Middleton Martin, the County Medical Officer of Health for Gloucestershire, gives valuable advice as to the best manner of developing schemes of treatment in connection with various groups of persons and classes of case for which provision has been made at the public expense. As he points out, it is easy in a county borough like Bristol, with 13,200 persons per square mile, but in Gloucestershire, with only 270 persons per square mile, it is impracticable to establish special centres for each class of case attended by a special medical officer in such a manner as to be accessible for inhabitants from every part of the county. On the other hand, there are administrative advantages in schemes worked by whole-time officers, and there are also undoubted advantages in securing the attendances of specialists for the examination and treatment of the special groups of persons. But unless the arrangements are such that all, or at least the greater number, of the persons resident in the area can attend at the centres, a considerable proportion of the population in country districts will be excluded by distance from these advantages. A further question arises. Are the advantages of specialists so great as to render their examination of all persons essential for the satisfactory working of a scheme? As to this, Dr. Martin says their experience with reference to the treatment of tuberculosis goes to show that a large part of the work of the tuberculosis officer is general consultant work on a wide variety of cases, presenting symptoms which may be due to tuberculosis, but which are often due to other conditions, and in practice he has to bring to bear not so much his specialist experience of tuberculosis as his whole general medical training. That the assistance of the specialist is necessary in every scheme there can be no doubt, but his services should be available in the way of consultation rather than in routine examination of all persons attending at the centres.

### The Lines of Organisation.

The requirements of county areas are :

1. Schemes of treatment must be so arranged that a centre is available for every part of the county, that is, the places of treatment must be generally distributed so that each class of case can have appropriate treatment even though the actual members in each group may and probably will be small.

2. Appropriate medical and nursing services must be available for each centre.

3. Arrangements must exist whereby consultant services may be readily and promptly available.

In the consideration of the practicability of making such arrangements, the question naturally arises whether or not there is existing machinery of which advantage can be taken in the development of schemes, and the answer is undoubtedly in the affirmative. In the large general hospitals are not only the most complete equipment for medical and surgical treatment but also the most highly-trained medical, surgical, and specialist services available in the whole country. Of these, there are four in or bordering on the county. There are, also, dotted about the county, eleven cottage hospitals, and, distributed over the county, 240 medical practitioners. In the general hospitals will be found the consultant services; and through the agency of the smaller hospitals and the medical practitioners, working closely in co-operation with the general hospitals, complete medical services available for the whole area of the county can be provided.

The need for considering the question of schemes of treatment for special groups has been brought out by :

1. The urgent need for making the scheme for the treatment of venereal diseases available, even for the scattered parts of the county.

2. The initiation of a scheme for the treatment of school children.

3. The desirability of increasing the facilities in connection with the tuberculosis scheme.

4. The requirements for the care of ex-Service men.

5. The developments in connection with maternity and child-welfare scheme, especially ante-natal work.

For all these provision has been made at the public expense, and there is strong probability that there will shortly be further developments, such as :

1. Extension of the medical services provided under the National Insurance Acts.

2. Transfer of the Poor-Law medical services to county councils, and their improvement and development.

### The Two Alternatives.

Two main courses offer themselves : One, an extension of the specialist services at present provided for tuberculosis and venereal diseases; and the other, the development of existing services and their improvement by close co-ordination. The necessity of adopting the latter course, if the advantages are to be available throughout the whole area of counties, has already been mentioned, and, if the co-operation between the medical practitioners and the consultants is complete—as it must be—not only will the schemes for the care of special classes be effective, but there will incidentally be important consequences in the direction of improving the efficiency of medical service, from the stimulus of the close association of all practitioners—specialist and general—with one another.

The initial difficulty in evolving a practical scheme on these lines is the number of bodies, central and local, affected, but in Gloucestershire a scheme giving effect to the proposals has been approved by all concerned—the county council, the general hospitals, the medical practitioners in the county at a meeting specially convened for the purpose, and by the Government departments.

Shortly, the scheme consists in the opening of some fifty out-stations, somewhat on the lines of existing tuberculosis dispensaries, with consulting-room, waiting-room, and dressing-rooms, so that one will be accessible within a radius of about every three miles of the county. At these, out-patient treatment of all the special classes of persons, for whom provision has at present been made at the public expense, or may be made in the future, will be available. The usual medical attendants will be the local practitioners, and the specialist staff of the hospitals will attend periodically for consultation and for the examination of cases reserved for them. An ambulance service is also proposed, which will be available for general hospital cases; but primarily the out-stations are intended for the special groups of cases where treatment will be chiefly such as can be given in an out-patient department.

The principal objection which has been raised is the mingling of the different classes of case in the same building, but owing to the large number of out-stations and the scattered populations served, there will be only one or two (or even none for some periods) of the special cases. It is, however, proposed that the utility of the out-stations shall be increased by making them available for any medical services (*e.g.*, out-patient treatment under the National Insurance Acts) by arrangement with the county council. In addition to medical services, intermediate treatment will also be given by nurses (school cases, etc.), trained orderlies (venereal diseases, etc.), masseurs and masseuses (ex-Service men, etc.), and it is hoped that arrangements may be made for the district nurses to reside at the out-stations.

Possibly more beds will be required for in-patient treatment, and these can be provided by enlarging existing hospitals and providing beds in suitable cases at out-stations as circumstances require and opportunity offers.

